# A deep learning method for translating 3DCT to SPECT ventilation imaging: First comparison with ^81m^Kr‐gas SPECT ventilation imaging

**DOI:** 10.1002/mp.15697

**Published:** 2022-05-17

**Authors:** Tomohiro Kajikawa, Noriyuki Kadoya, Yosuke Maehara, Hiroshi Miura, Yoshiyuki Katsuta, Shinsuke Nagasawa, Gen Suzuki, Hideya Yamazaki, Nagara Tamaki, Kei Yamada

**Affiliations:** ^1^ Department of Radiology Graduate School of Medical Science Kyoto Prefectural University of Medicine Kyoto Japan; ^2^ Department of Radiation Oncology Tohoku University Graduate School of Medicine Sendai Japan; ^3^ Department of Radiology Japanese Red Cross Kyoto Daini Hospital Kyoto Japan

**Keywords:** deep learning, functional imaging, radiotherapy

## Abstract

**Purpose:**

This study aimed to evaluate the accuracy of deep learning (DL)‐based computed tomography (CT) ventilation imaging (CTVI).

**Methods:**

A total of 71 cases that underwent single‐photon emission CT ^81m^Kr‐gas ventilation (SPECT V) and CT imaging were included. Sixty cases were assigned to the training and validation sets, and the remaining 11 cases were assigned to the test set. To directly transform three‐dimensional (3D) CT (free‐breathing CT) images to SPECT V images, a DL‐based model was implemented based on the U‐Net architecture. The input and output data were 3DCT‐ and SPECT V‐masked, respectively, except for whole‐lung volumes. These data were rearranged in voxel size, registered rigidly, cropped, and normalized in preprocessing. In addition to a standard estimation method (i.e., without dropout during the estimation process), a Monte Carlo dropout (MCD) method (i.e., with dropout during the estimation process) was used to calculate prediction uncertainty. To evaluate the two models’ (CTVI_MCD U‐Net_, CTVI_U‐Net_) performance, we used fivefold cross‐validation for the training and validation sets. To test the final model performances for both approaches, we applied the test set to each trained model and averaged the test prediction results from the five trained models to acquire the mean test result (bagging) for each approach. For the MCD method, the models were predicted repeatedly (sample size = 200), and the average and standard deviation (SD) maps were calculated in each voxel from the predicted results: The average maps were defined as test prediction results in each fold. As an evaluation index, the voxel‐wise Spearman rank correlation coefficient (Spearman *r*_s_) and Dice similarity coefficient (DSC) were calculated. The DSC was calculated for three functional regions (high, moderate, and low) separated by an almost equal volume. The coefficient of variation was defined as prediction uncertainty, and these average values were calculated within three functional regions. The Wilcoxon signed‐rank test was used to test for a significant difference between the two DL‐based approaches.

**Results:**

The average indexes with one SD (1SD) between CTVI_MCD U‐Net_ and SPECT V were 0.76 ± 0.06, 0.69 ± 0.07, 0.51 ± 0.06, and 0.75 ± 0.04 for Spearman *r*_s_, DSC_high_, DSC_moderate_, and DSC_low_, respectively. The average indexes with 1SD between CTVI_U‐Net_ and SPECT V were 0.72 ± 0.05, 0.66 ± 0.04, 0.48 ± 0.04, and 0.74 ± 0.06 for Spearman *r*_s_, DSC_high_, DSC_moderate_, and DSC_low_, respectively. These indexes between CTVI_MCD U‐Net_ and CTVI_U‐Net_ showed no significance difference (Spearman *r*_s_, *p* = 0.175; DSC_high_, *p* = 0.123; DSC_moderate_, *p* = 0.278; DSC_low_, *p* = 0.520). The average coefficient of variations with 1SD were 0.27 ± 0.00, 0.27 ± 0.01, and 0.36 ± 0.03 for the high‐, moderate‐, and low‐functional regions, respectively, and the low‐functional region showed a tendency to exhibit larger uncertainties than the others.

**Conclusion:**

We evaluated DL‐based framework for estimating lung‐functional ventilation images only from CT images. The results indicated that the DL‐based approach could potentially be used for lung‐ventilation estimation.

## 1 INTRODUCTION

Lung‐functional imaging has been reported to provide beneficial information for radiotherapy (e.g., functional lung‐avoidance radiotherapy treatment planning,^1^, ^2^, ^3^ and treatment response modeling^4^). Functional lung‐avoidance radiotherapy, especially, is used to irradiate tumors to minimize dose deposition to high‐functional lung regions by using guided functional imaging. The usefulness of this approach has been reported, and clinical practices and trials are underway.^5^, ^6^

Currently, most lung‐ventilation and ‐perfusion imaging methods are based on the use of radioisotopes or contrast media, such as single‐photon emission computed tomography (SPECT),^7^ positron emission tomography (PET),^8^ magnetic resonance imaging,^9^ and dual‐energy CT.^10^ Although these imaging techniques provide much beneficial information for regional lung function, they have some disadvantages, including radiation exposure, high cost, relatively low spatial and/or temporal resolution, and the requirement of special equipment. Therefore, the routine use of these methods for radiotherapy is difficult, so a more convenient approach for lung‐functional imaging is needed to perform functional lung‐avoidance radiotherapy. Recently, to overcome these shortcomings, image‐processing‐based methods have been proposed to quantify the local lung‐ventilation function from four‐dimensional (4D) CT images.^11^ This CT ventilation imaging (CTVI) technique is based on a deformable image registration algorithm that enables quantitation of the local lung‐ventilation function from 4DCT images acquired for treatment planning. Using this technique, the special medication or implementations mentioned previously are unnecessary, so acquiring the lung‐ventilation images becomes more convenient. Thus, 4DCT‐based ventilation imaging has high accessibility and the potential to be used in radiation oncology departments because no additional imaging and financial costs for the treatment protocol are required. In addition, studies on lung‐functional avoidance radiotherapy have reported the potential of substitutability for clinical ventilation imaging, such as SPECT^12^, ^13^ and PET,^8^, ^14^ and this has been used in some prospective clinical trials (NCT02528942 and NCT02843568). However, 4DCT results in higher radiation exposure and imaging cost compared with three‐dimensional (3D) CT.

In recent years, the use of deep learning (DL) for several tasks has become popular in radiotherapy, and particularly, convolutional neural networks (CNNs) have been primarily used for image processing.^15^ CNN consists of multilayered extraction and/or reconstruction features, and it is possible to perform end‐to‐end learning of features at various levels depending on the tasks. This technique has enabled CNN to provide comparable or superior performance to that of conventional approaches for various tasks, such as segmentation,^16^ dose prediction,^17^, ^18^ dose calculation,^19^ and quality assurance.^20^, ^21^ In addition, CNN was applied for lung‐functional image generation and provided performance superior to that of conventional CTVI techniques. Zhong et al. transformed inhale and exhale CT images in 4DCT images to lung‐ventilation images derived from CTVI directly.^22^ Liu et al. transformed 4DCT images to ventilation images derived from SPECT ventilation (V) directly.^23^ On the other hand, Ren et al. proposed and investigated a method for generating SPECT perfusion images directly from CT images and showed that the method could generate perfusion images relatively accurately only with 3DCT images.^24^, ^25^ Their results suggest that 3DCT images contain physiological and biological information necessary for estimating regional lung function.

Therefore, this study aimed to evaluate the accuracy of DL‐based approach for directly transforming 3DCT images to krypton‐81m gas (^81m^Kr‐gas) SPECT V images. The clinical SPECT V image was taken as true data, and the performance was evaluated qualitatively by visual inspection and quantitatively by indexes (Spearman rank's correlation coefficient and Dice similarity coefficient [DSC]). In addition, to interpret the prediction results, the prediction uncertainties were quantified and evaluated using the Monte Carlo dropout (MCD) approach.^26^ To the best of our knowledge, this is the first study to translate SPECT V images directly from 3DCT images.

## 2 MATERIALS AND METHODS

### 2.1 Patients and imaging

This study was a retrospective analysis approved by our Institutional Review Board (ERB‐C‐1811‐1). Sixty‐nine patients who underwent lung ^81m^Kr‐gas ventilation SPECT/CT scans from 2016 to 2021 at our hospital were enrolled. These patients had all the required data (e.g., attenuation‐corrected SPECT data and 3DCT images) for analysis. In total, 67 patients had 1 SPECT/CT image and 2 patients had 2 images (initial and follow‐up scan) acquired at a ≥6‐month interval from the initial SPECT/CT examination. The images of a total of 71 cases were used. Accordingly, 60 cases were assigned to the training and validation sets randomly, and the remaining 11 patients were assigned to the test set for dividing the initial and follow‐up scan in the same dataset to prevent data leakage. Among the 71 cases, 30 had pulmonary hypertension, 28 had pulmonary embolism, and 2 had systemic lupus erythematosus. The remaining patients had different types of lung diseases, such as paroxysmal supraventricular tachycardia and peripheral pulmonary stenosis. The clinical characteristics of the patients are summarized in Table 1.

**TABLE 1:** Clinical characteristics of the cases (*n* = 71): 67 patients had one SPECT/CT image set and 2 patients had 2 images

Characteristics	Median (range) or number (%)
Age (years)	72 (16–87)
Sex	Male: 18 (24.3%)/female: 53 (75.7%)
Smoking history	Yes: 20 (28.2%)/no: 50 (70.4%)/N/A: 1 (1.4%)
Diagnosis	Pulmonary hypertension: 30 (42.3%)
	Pulmonary embolism: 28 (39.4%)
	Systemic lupus erythematosus: 2 (2.8%)
	Others: 11 (15.5%)

*Note*: Others: paroxysmal supraventricular tachycardia, peripheral pulmonary stenosis, pulmonary arteriovenous fistula, atrial septal defect, severe tricuspid regurgitation, pulmonary vein stenosis, abdominal aortic aneurysm, transthyretin amyloid cardiomyopathy.

Abbreviations: CT, computed tomography; SPECT, single‐photon emission computed tomography.

We acquired ^81m^Kr‐gas ventilation images using a Discovery 670 SPECT/CT scanner (GE Healthcare, Milwaukee, Wisconsin). All scans were performed during the inhalation of 185 MBq of ^81m^Kr‐gas. The scans were acquired across 360° in six steps to cover the whole‐lung volume at a frame rate of 20 s/step. An extended low‐energy general‐purpose collimator was used, and attenuation correction was applied for ventilation scans. All SPECT images were collected as a 64 × 64 × 64 matrix with a pixel spacing of 8.84 × 8.84 × 8.84 mm^3^ using a 3D‐ordered subsets expectation maximization algorithm. The mean ± 1 standard deviation (1SD) of the non‐defect ventilation volume percentage segmented using the image‐specific intensity threshold (set at 50% of the 90th percentile value within the whole lung)^12^, ^14^ for all SPECT V images was 68.0% ± 11.1%.

All CT images were acquired on 64‐multidetector row CT scanners (Optima CT 540 or BrightSpeed; GE Health care, Milwaukee, Wisconsin) that compose the SPECT/CT system. All CT images were acquired during free‐breathing and collected as a 512 × 512 matrix with a pixel spacing of 0.977 × 0.977 mm^2^ in axial view with axial spacing of 1.25 mm and reconstructed using the same filtered back‐projection kernel for soft tissue. The X‐ray tube voltage and current were set to 75 kV and 120 mA, respectively. The radiation exposure of the CT technique with this scanner was 3.06 mGy in volume CT dose index.

### 2.2 Image preprocessing

**FIGURE 1:**
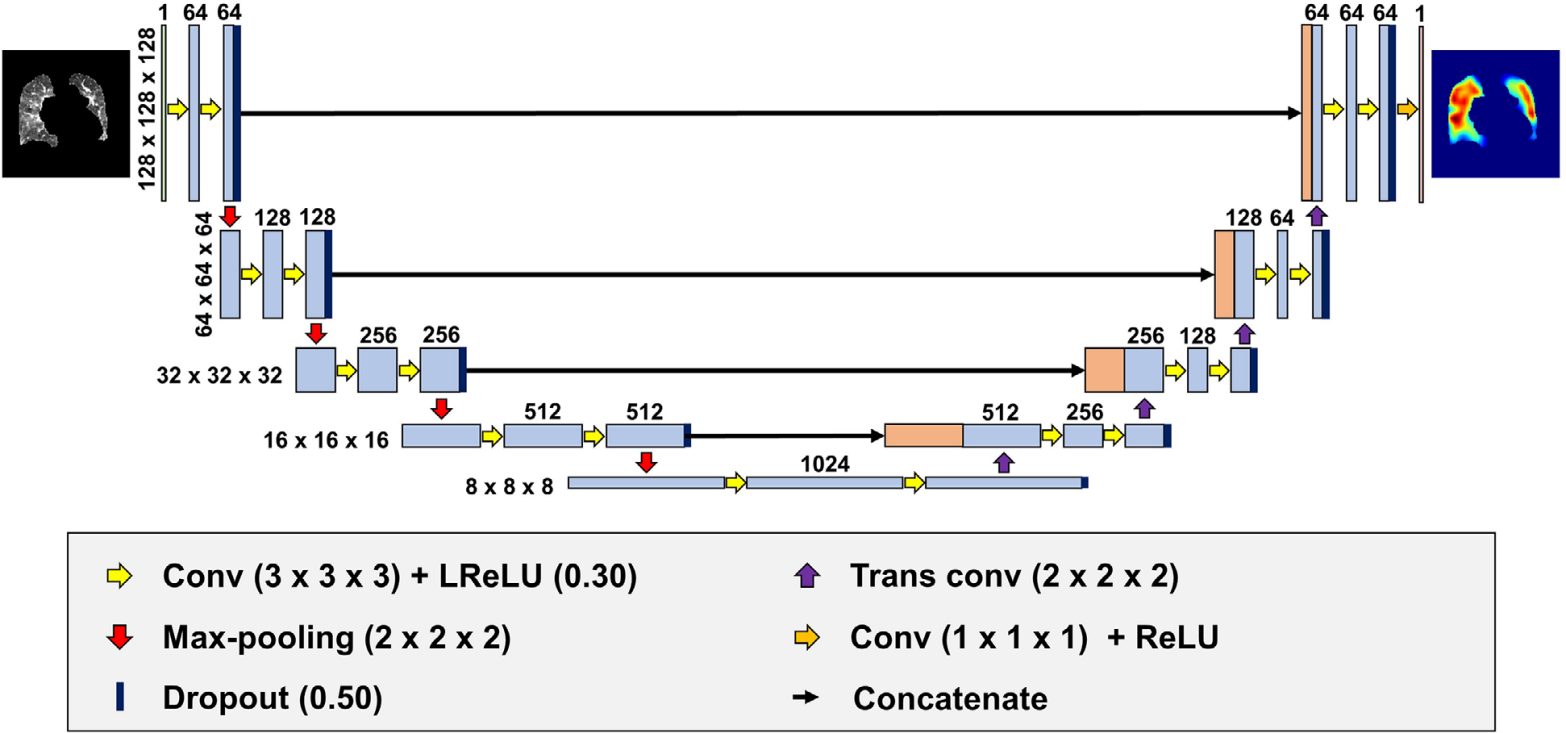
Model architecture used within the study. This CNN model is based on the U‐Net architecture. The masked CT volume is input into the model, and the masked ventilation volume is output through the encoding and decoding paths. Numbers on the left side and top of the model represent the volume shape and the number of feature maps in a particular layer, respectively. CNN, convolutional neural network; Conv, convolutional layer; CT, computed tomography; Trans conv: transposed convolutional layer

The pipeline of the image preprocessing for model training and evaluation consisted of the following processes: parenchyma segmentation, registration, pixel resampling and resizing, and rescaling or normalization. Lung parenchyma segments were automatically generated from the CT images using 3D slicer software (https://www.slicer.org: ver. 4.11) extension Chest Imaging Platform (http://www.chestimagingplatform.org) and modified manually if necessary (e.g., deleting obvious digestive gas). To minimize the influence of misalignment between the SPECT V and CT images, we registered the alignment rigidly on Elastix 5.0.1 using PyElastix 1.2 (https://github.com/almarklein/pyelastix) as the python wrapper. To reduce the computational cost in DL model training, the CT images and lung masks were down‐sampled and cropped into 2.5 × 2.5 × 2.5‐mm^3^ and 128 × 128 × 128‐sized matrices; these parameters were selected to include the whole‐lung volume in the input data for all patients. Then, the SPECT V images were up‐sampled and cropped under the same conditions as described previously. Voxel intensity values in CT are absolute and present important information (e.g., a single threshold of −856 HU indicates emphysematous lesions^27^). Therefore, to maintain the relationship along CT values, CT images were not standardized but rather rescaled linearly using the following scale to smoothly train the DL‐based model: −999 HU = 0 and −250 HU = 1. Conversely, voxel intensity values in SPECT imaging are relative rather than absolute compared with those in CT imaging, and it is possible that they have outliers (i.e., too high or too low local values). Therefore, to avoid the influence of outliers during normalization, SPECT V images were normalized to the median value, which is a relatively robust index, instead of the mean value of the whole‐lung volumes. These rescaled or normalized data volumes were replaced with 0 except for the whole‐lung volumes shown as the input and output in Figure 1. The previously mentioned processes were implemented using Python 3.8.8.

### 2.3 DL framework and training

We implemented a CNN model based on the U‐Net architecture.^28^ Figure 1 shows the model architecture. The architecture consists of encoding and decoding paths as with the general U‐Net architecture; the encoding path abstracts and extracts low–high‐dimensional features for input data, and the decoding path transforms the features to other domain output data. The encoding path consists of the repeated module of two 3 × 3 × 3 convolutions with 1 × 1 × 1 stride and 1 × 1 × 1 padding, each followed by a leaky rectified linear unit (Leaky ReLU)^29^ with alpha = 0.30 and a connecting dropout layer^30^ with a dropout rate = 0.50 and a 2 × 2 × 2 max‐pooling layer with a 2 × 2 × 2 stride for down‐sampling. At each down‐sampling step, we doubled the number of feature maps. Every step in the decoding path consisted of a 2 × 2 × 2 transposed convolutions with a 2 × 2 × 2 stride that halved the number of feature maps, a concatenation with the feature maps with corresponding size from the encoding path, and two 3 × 3 × 3 convolutions with a 1 × 1 × 1 stride and 1 × 1 × 1 padding, each followed by Leaky ReLU with alpha = 0.30 and a connecting dropout layer with a dropout rate = 0.50. At the final layer, a 1 × 1 × 1 convolution with a 1 × 1 × 1 stride followed by a ReLU^31^ was used for output. L2 regularization was set to 1.0 × 10^−4^ in all trainable layers. The 3D U‐Net was implemented in Keras with a TensorFlow 2.3.0 backend.

In addition to the standard estimation method, an MCD approach was used to quantify prediction uncertainty. In contrast to the standard method without dropout during estimation, the MCD approach approximates the Gaussian process by repeatedly sampling with dropout during the estimation phase of the model's weights from a Bernoulli distribution^26^ and could obtain a prediction uncertainty in each case.

To evaluate the model performance robustly, a total of 60 cases for training and validation were divided using a fivefold cross‐validation (CV) procedure: 48 cases were used for training and 12 cases were used for validation in each fold. To keep the study as fair as possible, the model architecture and datasets were fixed in both approaches (with or without MCD) during the training process. Adam^32^ (lr = 5.0 × 10^−5^, *β*_1_ = 0.90, *β*_2_ = 0.999, *ε* = 1.0 × 10^−7^) was used for the optimizer, the maximum train epoch was set to 1000, the batch size was set to 2, and early stopping (patience = 50) was used. We used the mean squared error (MSE) as the loss function. Computation was performed using an Intel Core i9‐9820X @ 3.30 Gz and an NVIDIA TITAN RTX GPU graphics card with a 24‐GB memory.

### 2.4 Evaluation

From these five trained models in each fold for both models with or without MCD, we then chose the best performance model on the basis of its validation loss (i.e., minimal error) and evaluated the model on the test set. To test these final model performances for the model without MCD (standard model), we applied the test set to each trained model and averaged the test prediction results from the five trained models to acquire the mean test result (bagging) for each approach. For the model with MCD (MCD model), the model was used to predict repeatedly (sample of size = 200), and the average and SDs calculated in each voxel from the predicted results were the prediction results for each fold.

For quantitative analysis, we calculated Spearman's rank correlation coefficient (Spearman *r*_s_) to assess the intensity correlation and the DSC to quantify the similarity of functional geometries for three separated nonoverlapping functional regions (i.e., high, moderate, and low) and MSE within the whole lung to evaluate the coincidence of raw intensity values. These functional regions were defined using the following thresholds: the highest 33.3 percentile value, lowest 33.3 percentile value, and between these values within the whole‐lung volume. These functional regions’ volumes were separated almost equally.

Spearman's *r*_s_ was defined by the following equation:

(1) $$r_{s}=\frac{\sum_{i=1}^{N}\left[\left(y_{i}-\bar{y}\right)\cdot \left(p_{i}-\bar{p}\right)\right]}{\sqrt{\sum_{i=1}^{N}{\left(y_{i}-\bar{y}\right)}^{2}}\sqrt{\sum_{i=1}^{N}{\left(p_{i}-\bar{p}\right)}^{2}}},$$

where $\bar{p}$, $\bar{y}$, $p_{i}$, and $y_{i}$ denote the average value and value at voxel *i* for the CTVI and SPECT V (ground truth), respectively. *N* denotes the total number of voxels in the whole lung.

DSC was defined by the following equation:

(2) $$DSC\left(a,b\right)=\frac{2\left|a\cap b\right|}{\left|a\right|+\left|b\right|},$$

where *a* denotes the volumes of functional lung regions in CTVI, and *b* denotes the volumes of the corresponding regions in SPECT V. Statistical differences were evaluated by the Wilcoxon signed‐rank test significant difference procedure using SciPy v1.6.3.

To compare the existing approach, we calculated DSC between SPECT V low‐functional regions and low‐attenuation (emphysematous lesion) areas derived from 3DCT. These low‐attenuation areas in 3DCT were defined as low‐functional regions using the single upper threshold limit of −856 HU.^27^ To keep the evaluation as fair as possible, SPECT V and CTVI images were segmented so that these volumes were almost equal to the low‐attenuation volumes in 3DCT in each case.

Furthermore, to evaluate the influence of misregistration between CT and SPECT V, we calculated the DSC between two masks generated from CT and SPECT V. The CT masks have been described previously. The SPECT V masks were generated from SPECT V images using a lower threshold limit of 15% of the maximum intensity value of the images,^33^ and they were modified manually if necessary (e.g., deleting obvious airway deposition). In addition, we evaluated Spearman *r*_s_ between the DSC calculated for these two masks and the quantification indices between SPECT V and CTVI.

For evaluating prediction uncertainty, SDs were calculated. Furthermore, to minimize the influence of large or small prediction values, the voxel‐wise coefficient of variation map was defined as the scaled uncertainty map, and the SDs were divided by the average values per voxel. For quantitative evaluation, the average values in three functional regions were calculated.

## 3 RESULTS

### 3.1 Fivefold cross‐validation

Table 2 summarizes the results of the voxel‐wise Spearman *r*_s_ and DSCs in the high‐, moderate‐, and low‐functional regions (DSC_high_, DSC_moderate_, DSC_low_, respectively) obtained between CTVI with MCD (CTVI _MCD U‐Net_) and SPECT V and between CTVI without MCD (CTVI _U‐Net_) and SPECT V within the whole‐lung volume for 11 test cases. For the average value of each fold, the average similarity metrics with 1SD between CTVI_MCD U‐Net_ and SPECT V were 0.73 ± 0.07, 0.67 ± 0.07, 0.48 ± 0.06, and 0.73 ± 0.05 for the Spearman *r*_s_, DSC_high_, DSC_moderate_, and DSC_low_, respectively. The average similarity metrics with 1SD between CTVI_U‐Net_ and SPECT V were 0.71 ± 0.06, 0.65 ± 0.04, 0.47 ± 0.04, and 0.73 ± 0.06 for the Spearman *r*_s_, DSC_high_, DSC_moderate_, and DSC_low_, respectively. Compared with CTVI_U‐Net_, CTVI_MCD U‐Net_ exhibited better performance in every evaluation index and showed comparable or superior performance in each fold with no statistically significant difference, excluding Spearman *r*_s_ and DSC_high_ in the second fold. Moreover, for the average value of each fold, the average MSE with 1SD values were 0.11 ± 0.04 and 0.15 ± 0.05 for CTVI_MCD U‐Net_ and CTVI_U‐Net_, respectively. Compared with CTVI_U‐Net_, CTVI_MCD U‐Net_ exhibited better performance in every evaluation index and showed superior performance in each fold with statistically significant difference, excluding those in the fifth fold.

**TABLE 2:** The prediction performance for the 11 test cases for each of the 5 models in each model with or without MCD (MCD U‐Net and U‐Net) trained by fivefold cross‐validation

			Dice similarity coefficient		
		Spearman *r*_s_	High	Moderate	Low
First fold	CTVI_MCD U‐Net_	0.73 ± 0.07	0.67 ± 0.08	0.48 ± 0.06	0.73 ± 0.05
	CTVI_U‐Net_	0.70 ± 0.06	0.64 ± 0.04	0.46 ± 0.04	0.73 ± 0.06
Second fold	CTVI_MCD U‐Net_	0.74 ± 0.05	0.68 ± 0.06	0.49 ± 0.05	0.74 ± 0.04
	CTVI_U‐Net_	0.70 ± 0.07	0.63 ± 0.04	0.45 ± 0.04	0.73 ± 0.06
Third fold	CTVI_MCD U‐Net_	0.74 ± 0.06	0.67 ± 0.07	0.48 ± 0.06	0.74 ± 0.05
	CTVI_U‐Net_	0.73 ± 0.05	0.65 ± 0.05	0.47 ± 0.04	0.75 ± 0.04
Fourth fold	CTVI_MCD U‐Net_	0.72 ± 0.07	0.67 ± 0.08	0.47 ± 0.06	0.72 ± 0.05
	CTVI_U‐Net_	0.71 ± 0.06	0.64 ± 0.04	0.46 ± 0.04	0.73 ± 0.06
Fifth fold	CTVI_MCD U‐Net_	0.73 ± 0.07	0.68 ± 0.06	0.48 ± 0.04	0.73 ± 0.05
	CTVI_U‐Net_	0.72 ± 0.06	0.66 ± 0.04	0.47 ± 0.04	0.73 ± 0.06
Average	CTVI_MCD U‐Net_	0.73 ± 0.07	0.67 ± 0.07	0.48 ± 0.06	0.73 ± 0.05
	CTVI_U‐Net_	0.71 ± 0.06	0.65 ± 0.04	0.47 ± 0.04	0.73 ± 0.06

Abbreviations: CTVI, computed tomography ventilation imaging; MCD, Monte Carlo dropout.

### 3.2 Bagging performance evaluation

**FIGURE 2:**
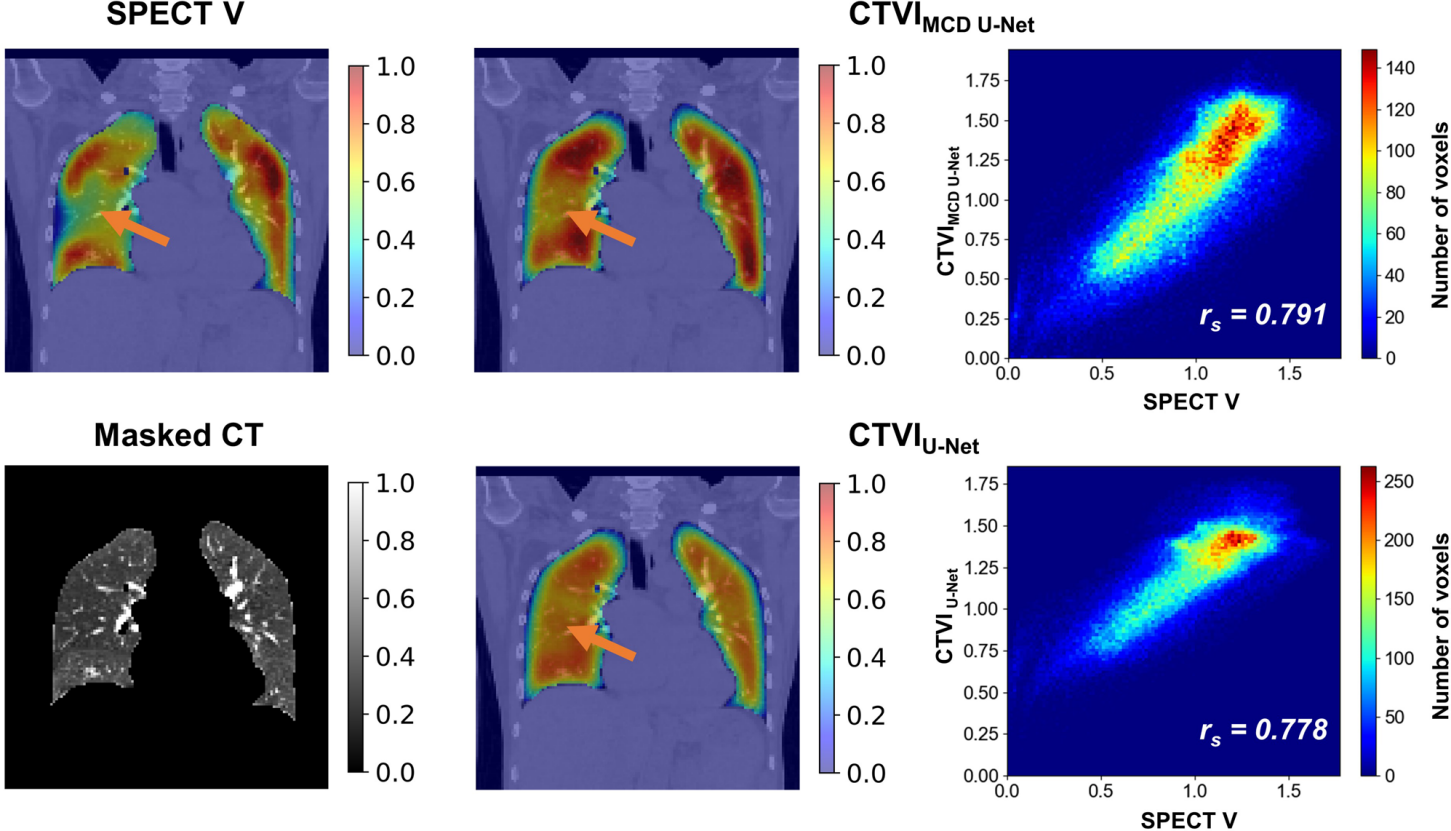
Typical cases of coronal slices showing relatively good performance based on the Spearman *r*_s_ values between SPECT V and CTVIs of both models. The top row shows the SPECT V on the left, followed by the ventilation predictions of the CTVI_MCD U‐Net_ and the scatter plot between SPECT V and CTVI_MCD U‐Net_ within whole‐lung volume. The bottom row shows the masked CT on the left, followed by the ventilation predictions of the CTVI_U‐Net_ and the scatter plot between SPECT V and CTVI_U‐Net_ within whole‐lung volume. Orange arrows indicate the defect regions of SPECT V. The ventilation values for viewing were normalized to give the 99th percentile value of 1 and the 1st percentile value of 0. CT, computed tomography; CTVI, computed tomography ventilation imaging; MCD, Monte Carlo dropout; SPECT V, single‐photon emission computed tomography ventilation

**FIGURE 3:**
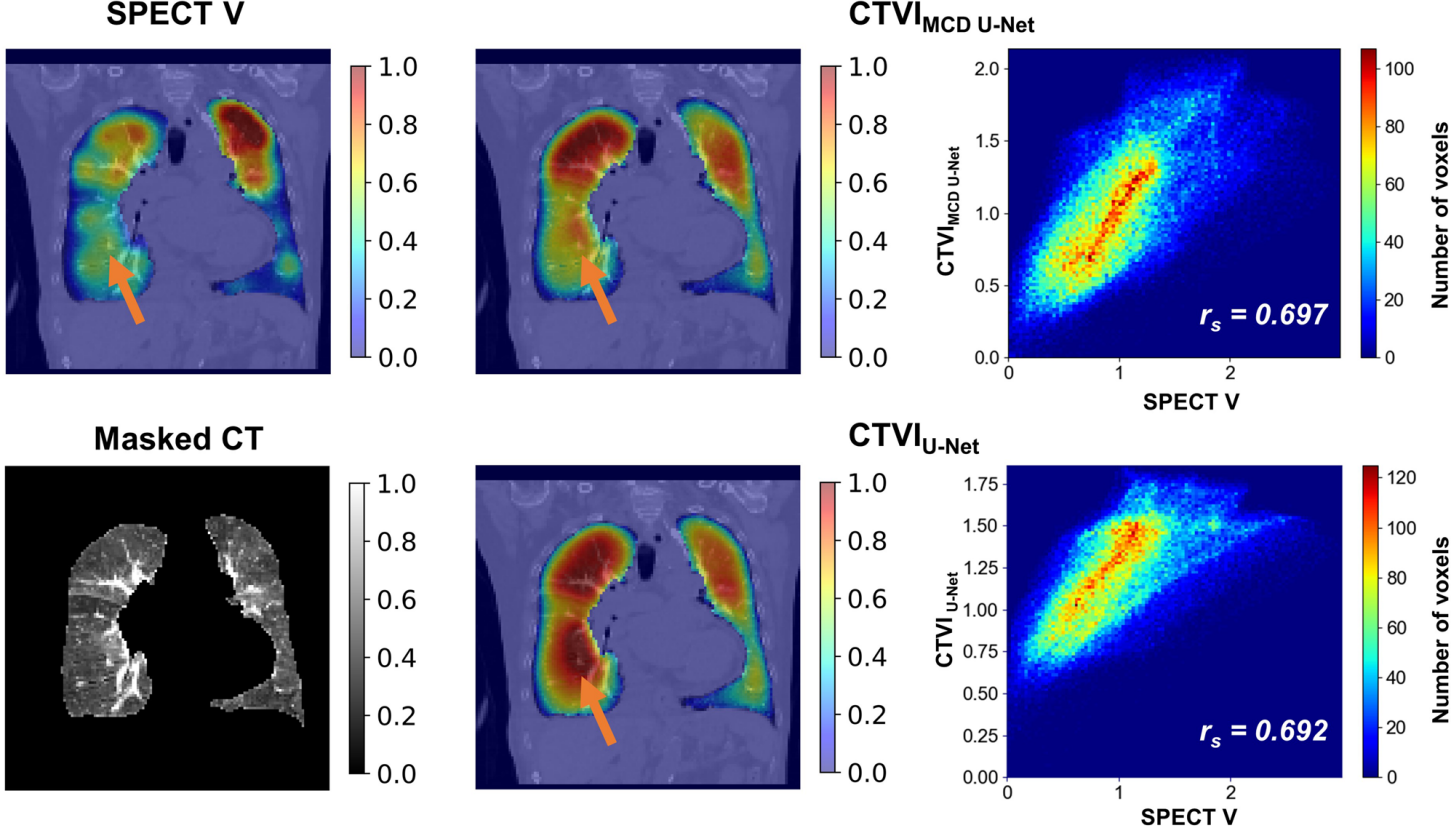
Typical cases of coronal slices showing relatively poor performances based on the Spearman *r*_s_ values between SPECT V and CTVIs of both models. The top row shows the SPECT V on the left side, followed by the ventilation predictions of the CTVI_MCD U‐Net_ and the scatter plot between SPECT V and CTVI_MCD U‐Net_ within whole‐lung volume. The bottom row shows the masked CT on the left side, followed by the ventilation predictions of the CTVI_U‐Net_ and the scatter plot between SPECT V and CTVI_U‐Net_ within whole‐lung volume. Orange arrows indicate the defect regions of SPECT V. The ventilation values for viewing were normalized to give the 99th percentile value of 1 and the 1st percentile value of 0. CT, computed tomography; CTVI, computed tomography ventilation imaging, MCD, Monte Carlo dropout; SPECT V, single‐photon emission computed tomography ventilation

Figures 2 and 3 show SPECT V, CTVI_MCD U‐Net_, CTVI_U‐Net_, masked CT coronal‐plane images, and scatter plots between SPECT V and each CTVI within whole‐lung volume for typical patients. The figures show two examples: one with relatively good agreement between DL‐based CTVI and SPECT V and the other with relatively poor agreement between DL‐based CTVI and SPECT V. Compared with CTVI_U‐Net_, CTVI_MCD U‐Net_ showed greater similarity with SPECT V. The low‐functional region (orange arrows) was predicted better by CTVI_MCD U‐Net_ than by CTVI_U‐Net_. Although most of the whole lung showed good agreement, both models with or without MCD tended toward functional overestimation.

**FIGURE 4:**
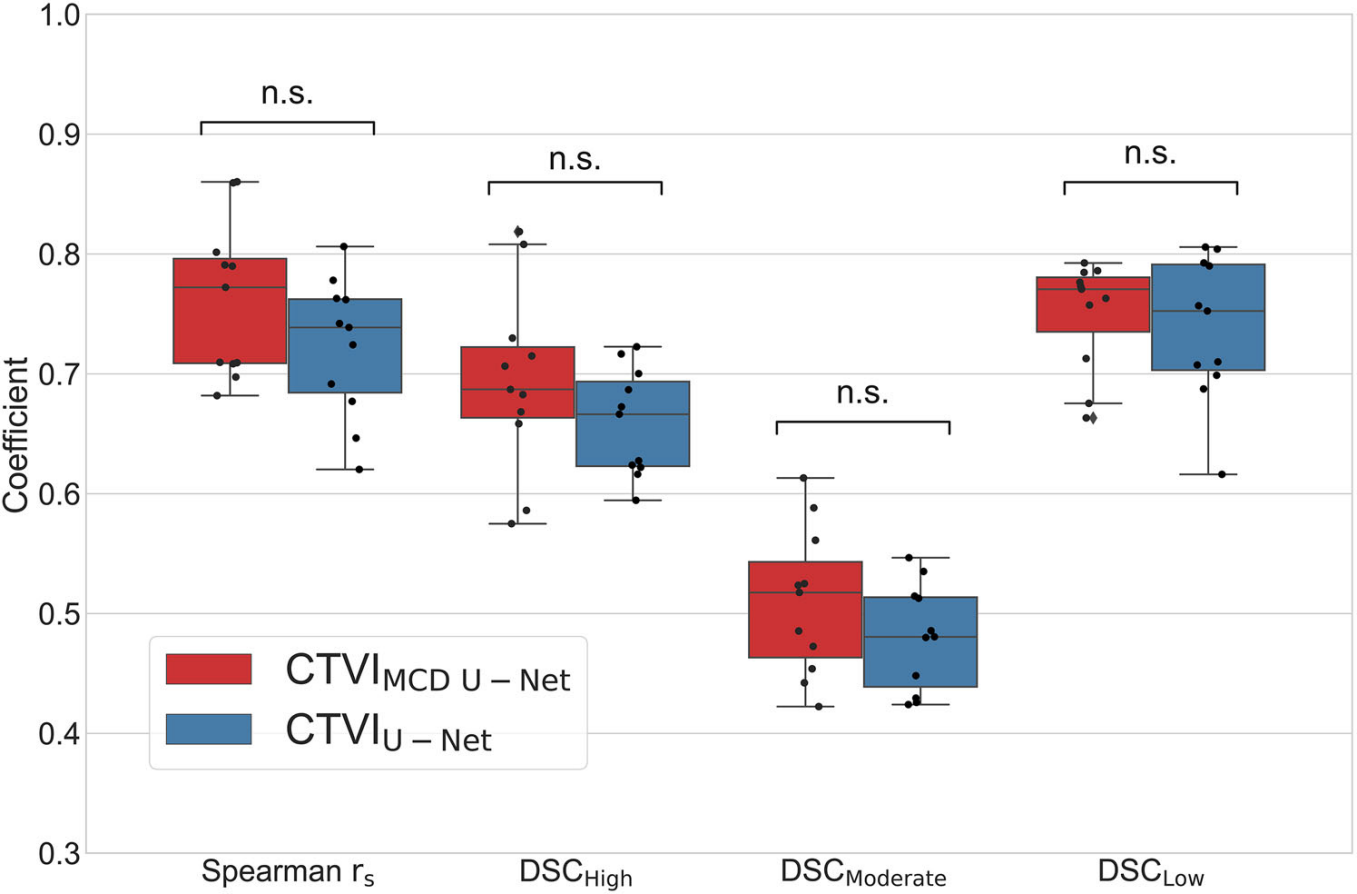
Boxplot and strip plot showing the bagged prediction performance for the 11 test cases for each of the 5 CNN models (MCD U‐Net and U‐Net) trained by fivefold cross‐validation. The Spearman *r*_s_ values are shown on the left, followed by the DSC_high_, DSC_moderate_, and DSC_low_ values between SPECT V and the two DL models. * *p* < 0.001. CNN, convolutional neural network; DSC, dice similarity coefficient; MCD, Monte Carlo dropout; n.s., not significant; SPECT V, single‐photon emission computed tomography ventilation

Figure 4 compares the Spearman rank correlation coefficients and DSCs for three functional regions between CTVI_MCD U‐Net_ and CTVI_U‐Net_ methods and SPECT V for the 11 test cases. The average similarity metrics with 1SD between CTVI_MCD U‐Net_ and SPECT V were 0.76 ± 0.06, 0.69 ± 0.07, 0.51 ± 0.06, and 0.75 ± 0.04 for the Spearman *r*_s_, DSC_high_, DSC_moderate_, and DSC_low_, respectively. The average similarity metrics with 1SD between CTVI_U‐Net_ and SPECT V were 0.72 ± 0.05, 0.66 ± 0.04, 0.48 ± 0.04, and 0.74 ± 0.06 for the Spearman *r*_s_, DSC_high_, DSC_moderate_, and DSC_low_, respectively. Compared with CTVI_U‐Net_, CTVI_MCD U‐Net_ had better performance with no statistically significant difference. Furthermore, the average MSE with 1SD values were 0.09 ± 0.04 and 0.12 ± 0.05 for CTVI_MCD U‐Net_ and CTVI_U‐Net_, respectively. Compared with CTVI_U‐Net_, CTVI_MCD U‐Net_ exhibited better performance with statistically significant difference. The DSC values for SPECT V were 0.48 ± 0.19, 0.47 ± 0.17, and 0.10 ± 0.07 for CTVI_MCD U‐Net_, CTVI_U‐Net_, and low‐attenuation areas in 3DCT, respectively. Therefore, compared with low‐attenuation areas, both CTVI_MCD U‐Net_ and CTVI_U‐Net_ showed better performance. Moreover, the Spearman *r*_s_ between DSC for the two masks (i.e., generated from CT or SPECT V) and the quantification indices between CTVI_MCD U‐Net_ and SPECT V were 0.19, 0.23, 0.35, and 0.02 for Spearman *r*_s_, DSC_high_, DSC_moderate_, and DSC_low_, respectively. Conversely, the Spearman *r*_s_ between DSC from these two masks and the quantification index between CTVI_U‐Net_ and SPECT V were 0.14, 0.32, 0.30, and −0.15 for Spearman *r*_s_, DSC_high_, DSC_moderate_, and DSC_low_, respectively. There was no tendency toward significantly worse prediction accuracy for the cases with relatively poor registration between CT and SPECT.

Table 3 summarizes the results of the voxel‐wise Spearman *r*_s_ values and DSCs in the high‐, moderate‐, and low‐functional regions obtained between the CTVI _MCD U‐Net_ and CTVI _U‐Net_ methods and SPECT V within the whole‐lung volume for 11 test cases for comparison with bagging and CV average results. The best performance of each result was CTVI_MCD U‐Net_ with bagging, followed by CTVI_MCD U‐Net_ with averaged CV results, CTVI_U‐Net_ with bagging, and CTVI_U‐Net_ with averaged CV results in descending order. The range of improvement of the bagging technique between the averaged CV results was larger for CTVI_MCD U‐Net_ than for CTVI_U‐Net_.

**TABLE 3:** Prediction performance for the bagging and fivefold CV averaging results for 11 test cases for each of the 5 CNN models (MCD U‐Net and U‐Net) trained by fivefold CV

			Dice similarity coefficient		
	Method	Spearman *r*_s_	High	Moderate	Low
CTVI_MCD U‐Net_	Bagging	0.76 ± 0.06	0.69 ± 0.07	0.51 ± 0.06	0.75 ± 0.04
	Average CV	0.73 ± 0.07	0.67 ± 0.07	0.48 ± 0.06	0.73 ± 0.05
CTVI_U‐Net_	Bagging	0.72 ± 0.05	0.66 ± 0.04	0.48 ± 0.04	0.74 ± 0.06
	Average CV	0.71 ± 0.06	0.65 ± 0.04	0.47 ± 0.04	0.73 ± 0.06

Abbreviations: CNN, convolutional neural network; CTVI, computed tomography ventilation imaging; CV, cross‐validation; MCD, Monte Carlo dropout.

### 3.3 Uncertainty evaluation

**FIGURE 5:**
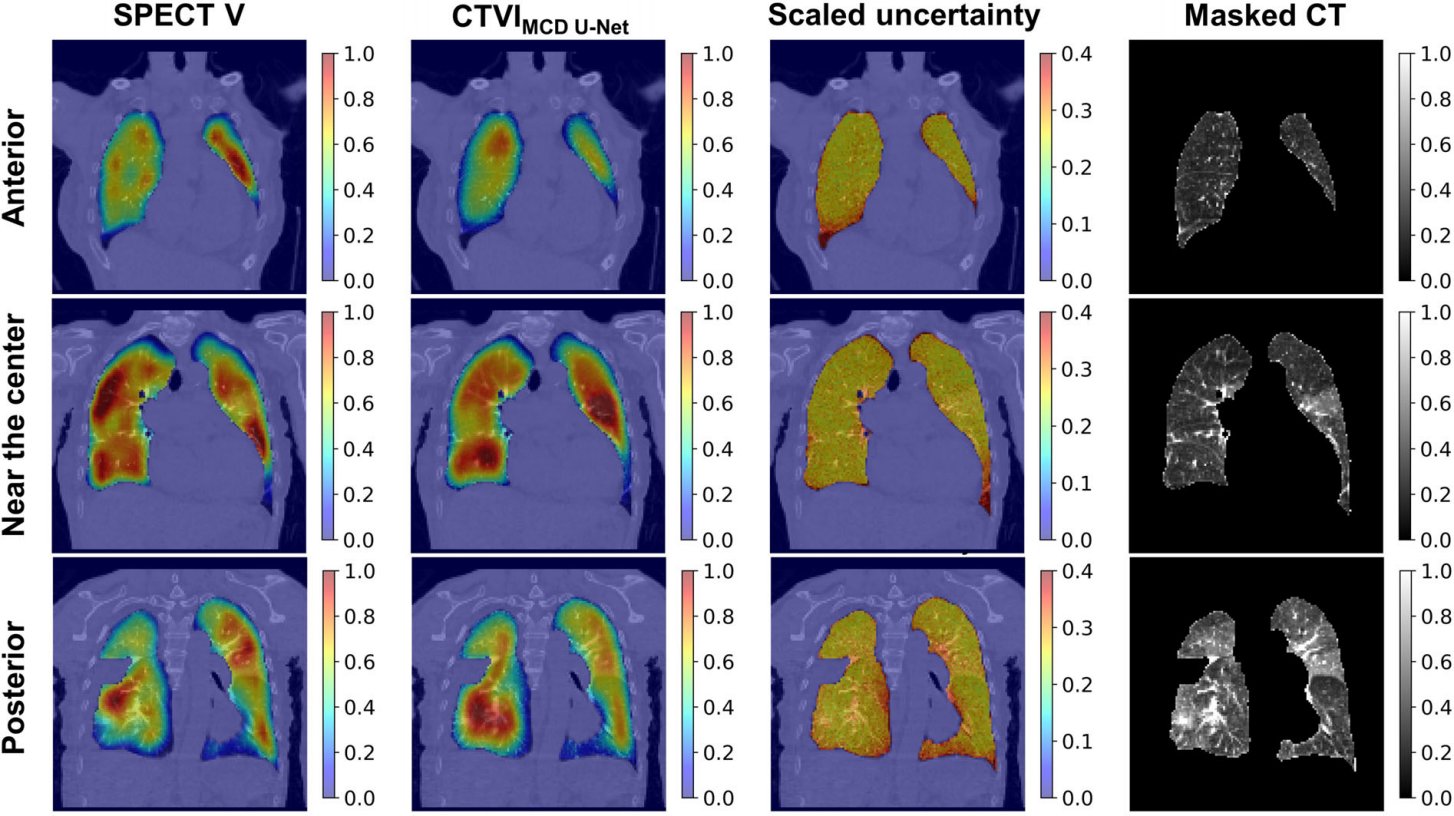
Typical cases of prediction of CTVI_MCD U‐Net_ in coronal slices. The anterior slice is shown on the top row, followed by the near, center, and posterior slices. The SPECT V is shown on the left, followed by CTVI_MCD U‐Net_, the scaled uncertainty for the CTVI_MCD U‐Net_, and masked CT. The ventilation values for viewing were normalized to give the 99th percentile value of 1 and 1st percentile value of 0. CT, computed tomography; CTVI, computed tomography ventilation imaging; MCD, Monte Carlo dropout; SPECT V, single‐photon emission computed tomography ventilation

Figure 5 shows the SPECT V, CTVI_MCD U‐Net_, scaled uncertainty, and masked CT images for a typical case in the coronal plane. The figure shows a tendency toward larger uncertainty on the lung edge (e.g., diaphragm) than in the center and larger uncertainty in the dorsal than ventral lung. The average SD values within the whole‐lung volume were 0.40 ± 0.01, 0.30 ± 0.01, and 0.19 ± 0.01 in the high‐, moderate‐, and low‐functional regions, respectively. Conversely, the average scaled uncertainties within the whole‐lung volume were 0.27 ± 0.00, 0.27 ± 0.01, and 0.36 ± 0.03 in the high‐, moderate‐, and low‐functional regions, respectively. Compared with the high‐ and moderate‐functional regions, the low‐functional region showed larger scaled uncertainty.

## 4 DISCUSSION

In this study, we evaluated the DL‐based framework that translates 3DCT images to lung‐ventilation images directly. Our DL‐based model had moderate or high performance, indicating that the DL‐based approach was effective for lung‐ventilation estimation mapping from 3DCT. To the best of our knowledge, this is the first study to evaluate the DL‐based approach for transforming 3DCT to SPECT V.

Our results showed that the DL approach had moderate or high performance for lung‐ventilation estimation; the average similarity metrics with 1SD between CTVI_MCD U‐Net_ and SPECT V were 0.76 ± 0.06, 0.69 ± 0.07, 0.51 ± 0.06, and 0.75 ± 0.04 for Spearman *r*_s_, DSC_high_, DSC_moderate_, and DSC_low_, respectively. Liu et al. implemented the DL‐based approach to transform 4DCT (two or ten phases) to ^99m^Tc‐gas SPECT V images.^23^ They showed that use of 10 phases had better performance than 2 phases. They showed that the average similarity metrics with 1SD when using 10/2 phases were 0.73 ± 0.16/0.71 ± 0.17, 0.83 ± 0.07/0.83 ± 0.07, 0.61 ± 0.10/0.59 ± 0.10, and 0.74 ± 0.10/0.72 ± 0.10 for Spearman *r*_s_, DSC_high_, DSC_moderate_, and DSC_low_, respectively. Although the trend of our results was similar to that of their results, our results were slightly inferior to theirs, which could have been caused by the following two factors: First, it is possible that 4DCT images contain more physiological and biological information necessary for estimating lung‐ventilation function than 3DCT images. Second, the differences in patient characteristics (patients with mainly circulatory disease vs. patients with esophagus or lung cancer) could have affected the results. Ren et al. implemented the DL‐based approach to transform 3DCT to SPECT perfusion images and investigated the influence of different model architectures and preprocessing.^24^, ^25^ They reported that there were some cases in which the low‐functional region was predicted to be a high‐functional region, which can be partly attributed to the low occurrence and small volume of the low‐functional regions compared with high‐functional regions. Our results showed similar trends (Figures 3 and 4). The percentage of non‐defect ventilation volume segmented and calculated in our SPECT V images was 68.0% ± 11.1%, and our datasets had larger non‐defect ventilation regions than defect regions. Thus, our DL model might have been affected by the distributions of ventilation regions and tended to the estimate low‐functional region to be a high‐functional region.

This study showed that CTVI_MCD U‐Net_ had better predictive performance with the bagging technique than CTVI_U‐Net_: 0.76 ± 0.06 versus 0.72 ± 0.05 for Spearman *r*_s_, 0.69 ± 0.07 versus 0.66 ± 0.04 for DSC_high_, 0.51 ± 0.06 versus 0.48 ± 0.04 for DSC_moderate_, and 0.75 ± 0.04 versus 0.74 ± 0.06 for DSC_low_. The DSC_moderate_ is always worse compared with DSC_high_ and DSC_low_. It could be caused by a technical issue in the evaluation method. We selected the evaluation approach with three nonoverlapping functional regions (i.e., high, moderate, and low) divided equally in volume based on threshold limits. This approach has generally been used in lung‐ventilation studies,^34^, ^35^ is suggested for use in functional lung‐avoidance radiotherapy treatment planning,^36^ and shows a similar tendency. In this approach, high‐ and low‐functional regions were defined by a single lower/upper threshold limit. Conversely, moderate‐functional regions were defined by two threshold limits. Therefore, moderate‐functional regions might have been more influenced by the border uncertainty compared with high‐ and low‐functional regions; therefore, the DSC value in moderate‐functional regions might be always the worst.

In addition, CTVI_MCD U‐Net_ showed an increased range of performance between bagging and fivefold CV averaged results compared to CTVI_U‐Net_: 0.03 versus 0.01 for Spearman *r*_s_, 0.02 versus 0.01 for DSC_high_, 0.03 versus 0.01 for DSC_moderate_, and 0.02 versus 0.01 for DSC_low_. The difference in performance could be caused by the difference in influences of the ensemble effect. The number of samples for estimation with the MCD model was set to 200 in each fold, bagging (i.e., averaging) the prediction results of 5 models from CV. Therefore, the influence of the ensemble effect was greater for the MCD model than for the standard model (1000 models vs. 5 models). Furthermore, these influences were observed for fivefold CV averaging results.

The scaled uncertainties were 0.27 ± 0.00, 0.27 ± 0.01, and 0.36 ± 0.03 in the high‐, moderate‐, and low‐functional regions, respectively. Compared with the high‐ and moderate‐functional regions, the low‐functional regions had larger scaled uncertainties. As mentioned previously, the low‐functional regions could not be estimated well because many patients showed normal function, and the number of patients showing deficits was small. In addition, as shown in Figure 5, the uncertainty tended to be large at the lung periphery and near the diaphragm. The blurring around the diaphragm was particularly large and could have been caused by breathing and heartbeat as well as by the variability in lung shapes among the patients. The blurring problem might be mitigated by aligning the sizes of pulmonary parenchyma in SPECT and CT images well. Furthermore, the improvement of SPECT images using respiratory‐gated motion correction^37^ during image acquirement might be effective.

Although our results showed the potential of DL‐based approach for lung‐ventilation estimation mapping, there were several limitations in this study. First, our datasets have bias in patient background. Generally, DL is strongly affected by the bias of the datasets. Although our results showed that the DL‐based model had moderate or high performance in this study, it is unknown whether the models trained by our datasets can be adapted for those who are undergoing radiotherapy and different patient backgrounds. Our datasets were different from clinical population receiving radiotherapy generally (e.g., in terms of non‐defect ventilation volume and gender). The percentage of the non‐defect ventilation volume was 68.0% ± 11.1%, and the datasets had a larger percentage of non‐defect ventilation volume than the clinical population with early‐stage primary non–small‐cell lung cancer (53.7% ± 15.5%) in the previous study.^12^ This occurred because our datasets consisted of cases showing non‐defect ventilation regions (e.g., pulmonary hypertension and pulmonary embolism). In addition, the datasets had gender imbalance because of the bias of the clinical population in our hospital patient cohort, as it is different from the clinical population (male > female) receiving radiotherapy treatments such as for lung cancer. Further studies are necessary to investigate the effect of data characteristics, and we will expand the data pool with more datasets. Second, the model architecture and hyper parameters were not completely optimized for the lung‐ventilation estimation mapping task. In this study, the model architecture was based on the standard U‐Net model for image generation in medical imaging, and the hyper parameters were set to general parameters (i.e., optimizer: Adam, dropout rate: 0.50). Although we only used a relatively general model and hyper parameters, we did not consider any optimization, such as adding layers or tuning hyper parameters. Therefore, the optimization of the model architecture and hyper parameters may improve the estimation performance. Third, positional errors between SPECT V and 3DCT were observed in our dataset. Although the 3DCT and SPECT V images were acquired during the same examination series, differences in patient positions and/or anatomical geometries (e.g., lung shape and diaphragm position) between SPECT V and 3DCT were observed because of changes in the breathing patterns during SPECT and/or 3DCT imaging. To minimize the influence of these errors, we registered SPECT V and 3DCT images rigidly at preprocessing, and the registration accuracy for each patient was checked visually. However, the influences of changes in breathing patterns between the SPECT V and 3DCT images were not completely eliminated, and there was some possibility that data were affected in the training and/or prediction process by these mismatches. Further studies are necessary to investigate the influence of these mismatches. Finally, the approach for uncertainty quantification employed in this study was the simplest method (i.e., calculating only the SD). Therefore, we did not calibrate the quality of uncertainty quantification and only calculated SDs as epistemic uncertainty (not aleatoric uncertainty). Although this approach has been used in previous medical‐imaging studies,^38^, ^39^ it has some limitations as described previously. Further studies are necessary to estimate more sophisticated uncertainty quantification for DL‐based frameworks that translate lung‐ventilation images from 3DCT images.

This pulmonary ventilation mapping prediction tool might be useful for adaptive lung‐functional avoidance radiotherapy planning. Yuan et al. reported that changes in pulmonary regional ventilation and perfusion functions were sometimes observed by SPECT V/Q during the course of radiotherapy because of tumor shrinkage.^40^ Kipritidis et al. also reported inter‐fractional changes in pulmonary regional ventilation functions captured by CTVI based on cone‐beam (CB) CT over a course of radiotherapy, supporting the feasibility for adaptive lung‐ventilation‐avoidance radiotherapy.^41^ Our results showed that the DL‐based approach was effective for lung‐ventilation estimation mapping from CT. Therefore, this approach could be potentially applied for CBCT to lung‐ventilation translation. CBCT is often acquired for positional alignment during the course of radiation treatment, and it might be possible to monitor these inter‐fractional pulmonary functional changes in a simpler way. This DL‐based approach could also be potentially used for adaptive functional lung‐avoidance radiotherapy and treatment response modeling.

## 5 CONCLUSIONS

We evaluated the performance and uncertainty of DL‐based frameworks that translated 3D lung‐ventilation images from 3DCT images. Our results suggested that the DL‐based approach was effective for lung‐ventilation estimation mapping from 3DCT.

## CONFLICT OF INTEREST

The authors declare that they have no known competing financial interests or personal relationships that could have appeared to influence the work reported in this paper.
